# A novel technique for the treatment of infected metalwork in orthopaedic patients using skin closure over irrigated negative pressure wound therapy dressings

**DOI:** 10.1308/003588413X13511609957254

**Published:** 2013-03

**Authors:** R Norris, AWP Chapman, S Krikler, M Krkovic

**Affiliations:** University Hospitals Coventry and Warwickshire NHS Trust, UK

**Keywords:** Vacuum-assisted closure, Negative pressure wound therapy, Infected orthopaedic metalwork

## Abstract

**Introduction:**

There has been recent interest in the use of negative pressure wound therapy (NWPT) as an adjunct to parenteral antibiotics in the treatment of infection in orthopaedic patients with metalwork in situ. To address some of the limitations of standard NPWT in this situation, the senior author has developed a modified method of treatment for infected metalwork (excluding arthroplasty) in orthopaedic patients that includes irrigation and skin closure over the standard NPWT dressing.

**Methods:**

This retrospective study examined the outcome of a case series of 16 trauma and orthopaedic patients with deep infection involving metalwork in whom this modified form of NPWT was used. In conjunction with standard parenteral antibiotic therapy and a multidisciplinary approach, this modified technique included serial debridements in theatre, irrigation and negative pressure dressings over a white polyvinyl alcohol foam (KCI, Kidlington, UK) as well as closure of the skin over the foam.

**Results:**

Among the 16 patients, there was a variety of upper and lower limb as well as spinal trauma and elective cases. In all 16 patients, there was successful resolution of the infection with no early or unplanned removal of any metalwork required.

**Conclusions:**

Patients with infected metalwork are a heterogeneous group, and often suffer high morbidity and mortality. The modified NPWT technique shows potential as an adjunct in the treatment of complex orthopaedic patients with infected metalwork.

Despite many prophylactic measures, infection of the implant occurs in 1–12% of orthopaedic patients operated on for arthroplasty or osteosynthesis.^1^ Patients with infected metal implants suffer significant morbidity and mortality, and treatment of infection in relation to metalwork is usually a prolonged and expensive process, with significant impact on a patient’s quality of life and health. There may be many contributing factors to the development and persistence of infection, eg medical co-morbidity, host immuno-suppression, poor tissue viability and the type of metalwork involved. At present, there is no gold standard agreed for treatment of these patients and, in our unit, these patients are generally managed by a multidisciplinary team (MDT) approach including orthopaedic surgeons and microbiologists.

More recently, negative pressure wound therapy (NPWT) dressings have been used to allow retention of the metalwork while still curing the infection. This therapy is not a substitute for established practice but should be seen as an adjunct and may provide a valuable alternative when retention of the metalwork is desired. The standard principles of an MDT approach, debridement, irrigation and microbiology sampling prior to commencing antibiotic treatment are still essential in the treatment of these cases.

NPWT dressings are a form of NPWT and consist of three main components. A specialised foam dressing is placed into the wound and sealed with adhesive dressings. A tube is attached that connects to a pump, which then provides continuous or intermittent negative pressure, and allows monitoring and regulation of the pressure inside the wound. NPWT dressings are reported to promote wound healing by reducing oedema and toxins, reducing the bacterial load, stimulating angiogenesis and bringing the wound edges closer together.^2^

VAC Instill^®^ (KCI, Kidlington, UK) refers to conventional NPWT combined with a continuous perfusion or irrigation system and has been shown to be effective in treating wounds with very high bacterial burdens.^3^ The VAC Instill^®^ option offers the possibility of regular cleansing of foam sponges (which prolong their life and therefore reduce the required frequency of dressing changes) and enables continuous irrigation of the wound with fluid, which may help to decrease the overall bacterial load. The major disadvantage of VAC Instill^®^ in the context of infected metalwork is that the exposed metalwork is only covered by the NPWT sponge and transparent adhesive dressings, and may be prone to leaking, loss of pressure or secondary infection if the seal is not maintained.

In an attempt to overcome these difficulties, we changed our working practice with regard to the use of NPWT dressings in patients with infected metalwork. The senior author (MK) has developed a modified method of NPWT for patients with infected metalwork using a combination of the irrigation NPWT and wound closure to allow the metalwork to remain in situ while the infection is being treated.

The advantages of this are that direct wound closure over the NPWT dressing creates the most effective barrier to infection, and prevents skin contracture and heat loss in large wounds. This method also allows continuous irrigation, which has been found to be advantageous in the VAC Instill^®^ regime.^3^ Furthermore, this technique deals with the significant problem of leakage with the irrigation treatment and the loss of vacuum as well as maintaining wound and metalwork sterility with it only being exposed during wound debridement in the operating theatre.

## Methods

A retrospective review was carried out on all patients treated by a single orthopaedic consultant with the modified NPWT as part of their surgical treatment for infection between 2008 and 2010. As the aim of this study was to review the use of the modified technique in patients with metal work in situ only, patients were excluded from review if there was no metalwork in situ, if it was removed prior to the NPWT commencing or if they had an arthroplasty or hemiarthroplasty (as different treatment protocols exist in patients who have undergone arthroplasty-type procedures). Patients were also excluded if they did not tolerate the NPWT, if they were transferred to another hospital during treatment or if they were not fit enough to complete the treatment.

All patients were also treated with antibiotics targeted to their infective organism, from samples taken during the first debridement. There was no standardised antibiotic formulary due to the heterogenic nature of the patients and each patient’s antibiotic regimen was decided based on microbiologist advice.

The patients’ electronic and paper medical records were reviewed. The data collected included information on the type of metal implant involved, whether the infection occurred early in the postoperative course (<6 weeks postoperatively) or late (>6 weeks), the demographics of the patients, the microbiology involved and whether they were elective or trauma patients. The primary endpoint was whether the patient retained his or her metal implant with resolution of the infection. The infection was considered resolved if there were no clinical signs of infection and infection parameters were normal, including C-reactive protein (CRP), erythrocyte sedimentation rate (ESR) and white cell count (WCC). As per standard practice in patients with infected metalwork, the patients in this case series underwent routine examination and investigations including blood parameters for infection (CRP, ESR, WCC and blood cultures) as well as any appropriate imaging.

All patients went to the operating theatre for an initial thorough and complete debridement of the infected tissue under general anaesthesia (GA), leaving the metalwork in situ. Tissue and fluid samples are also taken at this time for microbiological analysis. The VAC^®^ system was used for this study. The VAC^®^ dressing was then applied to the wound in the standard fashion directly onto the metalwork and tissue without any lining but, for this modified technique, a small pore non-adherent white polyvinyl alcohol foam (KCI, Kidlington, UK) was substituted for the wide pore black polyurethane foam (GranuFoam™; KCI, Kidlington, UK). The hydrophilic white polyvinyl alcohol foam is advantageous as the bacterial load counts are known to be lower than with the black polyurethane foam and the foam can therefore be left in for longer periods of time, which decreases the number of visits to the operating theatre.

**Figure 1:**
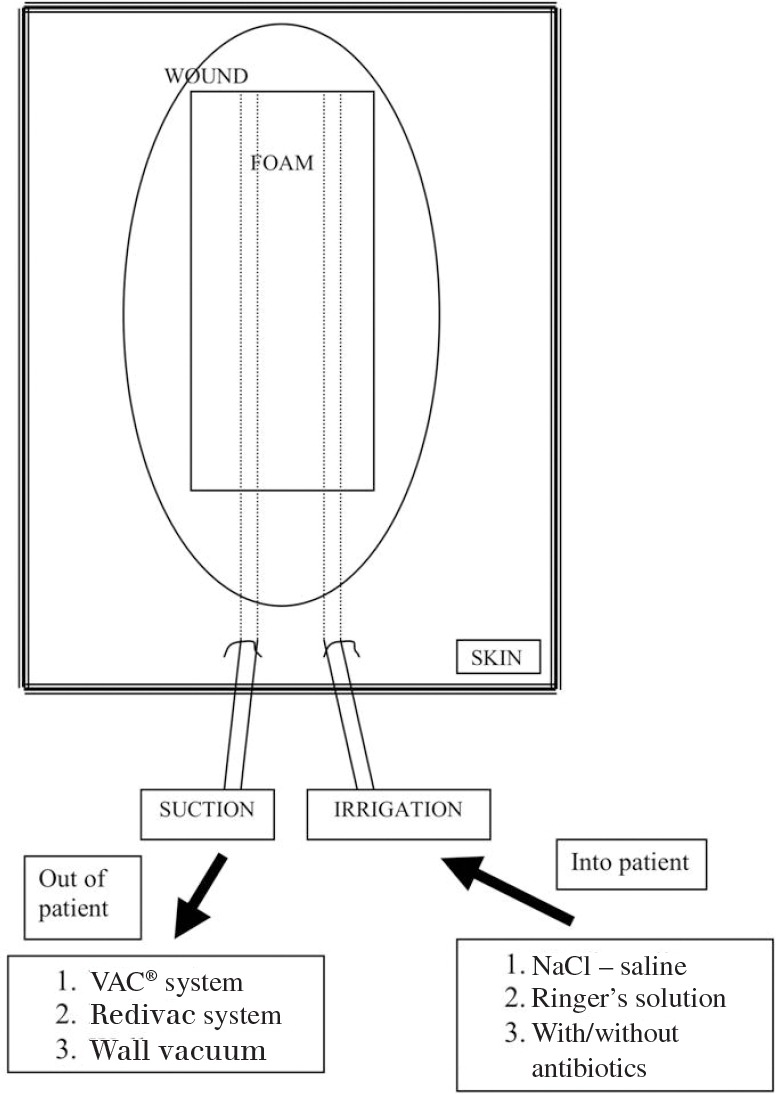
Negative pressure wound therapy irrigation arrangement

In addition to the white foam, two Redivac drains (Biomet, Bridgend, UK) were led out through the skin with the help of a trocar (Fig 1). A sterile trocar from the Redivac drain works well for this purpose, and we prefer this technique to a skin incision for the insertion of the drain as this leads to leakage and loss of vacuum. One drain was attached to the vacuum source (theatre suction system) at 125mmHg and the other to a bag of normal saline to provide irrigation. Normal saline is the fluid of choice for irrigation. Ringer’s lactate or glucose solutions should be avoided to prevent bacterial overgrowth. Irrigation and suction were applied as soon as possible after the foam was placed in situ into the wound to prevent formation of blood clots on the foam.

**Figure 2:**
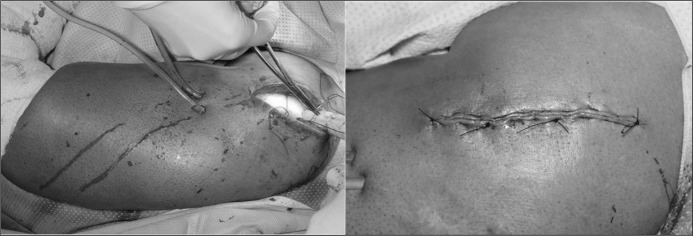
Fascial and skin closure over the negative pressure wound therapy dressing

The wound was closed in layers (over the VAC^®^ foam) with interrupted absorbable synthetic braided sutures to the deeper fascial layers and subcutaneous tissue, and a continuous synthetic non-absorbable nylon suture for the skin (Fig 2). If the subcutaneous tissue was also infected, a separate piece of foam was applied to this layer. After wound closure, the suction drain was attached to a VAC^®^ machine (or wall suction) on the continuous setting. The fluid was set to run in at a rate of 2l every 24 hours, which provided continual cleansing to the wound and foam as shown in Figure 1.

**Figure 3:**
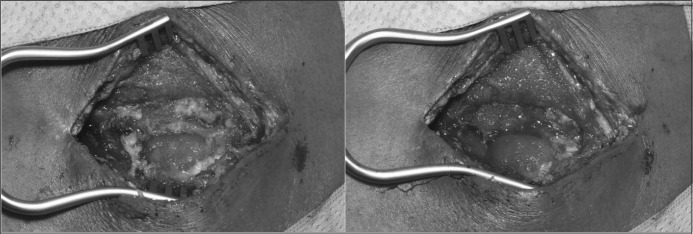
Difference between the initial debridement (left) and the second look (right)

The VAC^®^ dressing was left in situ for approximately 6–8 days. The patient was then taken back to the operating theatre under GA for a second debridement and change of the dressing. At this stage, it was possible to distinguish clearly between vital (covered with granulation tissue) and necrotic tissue (Fig 3). The VAC^®^ dressing was reapplied as described above but the sponges were slightly undersized to accommodate for wound shrinkage as the infection is treated and the tissues heal. This process was repeated every 6–8 days until the wound looked clean, with no necrotic tissue, and the infection parameters had normalised.

Once the resolution of infection was achieved, the VAC^®^ dressing was removed completely under GA, including all sponge debris, and the wound was closed in a standard fashion with a Redivac drain left in situ for 72 hours, which was later removed on the ward. Most patients required three visits to theatre under GA: one for the initial debridement, one for a second debridement and smaller foam insertion, and a third for closure.

## Results

The 60 patients who underwent this modified NPWT in our department during the study period for any reason were recorded. The reasons for the therapy are shown in Table 1. Of these patients, a subgroup of 16 (9 male, 7 female) had NPWT in the presence of infected metalwork. Of this subgroup, the mean patient age was 42 years (range: 17–72 years). There were ten trauma and six elective patients. Thirteen of the infections were of early onset and three were of late onset. There were five spinal cases, five upper limb cases and six lower limb cases. Patients required a mean of 2.8 visits (mode: 2) to theatre to have the dressing changed or removed after the initial debridement. Each of the 16 patients retained his or her implant with complete resolution of the infection. The exact details of each group are shown in Tables 2 to 4.

**Table 1:** All patients undergoing negative pressure wound therapy (NPWT)

Reason for therapy	Number of patients
Infections with metalwork in situ	16
Open fractures with tissue loss but no infection	11
Soft tissue cases with no metalwork involved, most of which had no infection present (compartment syndrome, septic arthritis of the shoulder, muscle repair, spinal decompression)	10
Metalwork removed prior to the use of NPWT	10
Infected arthroplasty/hemiarthroplasty	9
Died of an unrelated cause before completion of treatment	2
Transferred to another hospital during treatment	1
Did not tolerate NPWT	1

**Table 2:** Spinal group results

Patient number	Patient age	Early or late infection	Procedure	Time after first washout that NPWT was applied	Duration of NPWT	Number of NPWT changes	Long-term outcome
1	17 years	Late	Corrective surgery for severe scoliosis	0 days	21 days	3	Metalwork retained, no infection
2	46 years	Early	Instrumented lumbar spine fusion	0 days	14 days	2	Metalwork retained, no infection
3	43 years	Early	Instrumented lumbar spine fusion	0 days	14 days	2	Metalwork retained, no infection
4	41 years	Early	Instrumented anterior lumbar spine fusion	0 days	15 days	2	Metalwork retained, no infection
5	72 years	Early	Instrumented lumbar spine fusion	0 days	14 days	2	Metalwork retained, no infection

NPWT = negative pressure wound therapy

**Table 3:** Upper limb group results

Patient number	Patient age	Early or late infection	Procedure	Time after first washout that NPWT was applied	Duration of NPWT	Number of NPWT changes	Long-term outcome
6	28 years	Early	Open shoulder stabilisation (Latarjet)	12 days	15 days	2	Metalwork retained, no infection
7	64 years	Early	Proximal humeral nail	0 days	28 days	4	Metalwork retained, no infection, fracture union
8	65 years	Early	ORIF proximal humerus (PHILOS plate)	4 days	14 days	2	Metalwork retained, no infection, fracture union
9	39 years	Early	ORIF greater tuberosity	0 days	15 days	2	Metalwork retained, no infection, fracture union
10	24 years	Early	Hook plate for ACJ disruption	2 days	12 days	2	Metalwork retained, no infection (has since had plate removed; routine for type of implant)

NPWT = negative pressure wound therapy; ORIF = ORIF = open reduction internal fixation; PHILOS = Proximal Humeral Interlocking System; ACJ = acromioclavicular joint

**Table 4:** Lower limb group results

Patient number	Patient age	Early or late infection	Procedure	Time after first washout that NPWT was applied	Duration of NPWT	Number of NPWT changes	Long-term outcome
11	19 years	Early	Infection after tibial plateau fracture fixation	0 days	36 days	5	Metalwork retained, no infection, fracture union
12	50 years	Early	Total hip replacement periprosthetic fracture ORIF	3 days	27 days	4	Metalwork retained, no infection, fracture union
13	45 years	Early	Abscess after ankle fusion	0 days	15 days	2	Metalwork retained, no infection, metalwork removed electively 3 months after union
14	61 years	Late	Wound breakdown after ankle ORIF	2 days	39 days	5	Metalwork retained, no infection, fracture union
15	32 years	Late	Abscess 2 months into treatment of open fracture with external fixation as definitive fixation	0 days	33 days	4	Treated with external fixation, no infection, fracture union
16	22 years	Early	Infection after tibial ORIF	0 days	14 days	2	Metalwork retained, no infection, fracture union, plate removed electively at a later date

NPWT = negative pressure wound therapy; ORIF = open reduction internal fixation

The most common infective organisms were *Staphylococcus aureus*, coliforms and *Enterococcus* spp. Overall, there were 9 different infective organisms in the 16 patients. Each patient’s antibiotic regimen was tailored to his or her specific requirements based on the microbiologists’ advice. Full details of the infective organisms and the antibiotics used are shown in Tables 5 and 6.

**Table 5:** Microbiology summary of infections

Causative organism	Organism incidence	Antibiotics used
*Staphylococcus aureus*	6	Flucloxacillin, rifampicin, doxycycline, clindamycin and co-amoxiclav
*Enterococcus* spp	5	Flucloxacillin, gentamicin, clindamycin, metronidazole, vancomycin, rifampicin and meropenem
Coliform	3	Flucloxacillin, rifampicin, clindamycin, doxycycline and meropenem
MRSA	2	Vancomycin, rifampicin, gentamicin, mupirocin and chlorhexidine
Coagulase-negative *Staphylococcus*	2	Flucloxacillin, meropenem, vancomycin and metronidazole
*Proteus* spp	1	Flucloxacillin, piperacillin/tazobactam, vancomycin and rifampicin
*Klebsiella pneumoniae*	1	Flucloxacillin and rifampicin
Skin flora only	1	Vancomycin, rifampicin, flucloxacillin and fusidic acid
Group A *Streptococcus*	1	Co-amoxiclav

**Table 6:** Detailed microbiology of infections

Patient number	Causative organism	Antibiotics used (length of course)
1	Coagulase-negative *Staphylococcus* and *Enterococcus* spp	Meropenem and vancomycin (4 weeks), then metronidazole (4 weeks)
2	*Staphylococcus aureus*	Rifampicin, metronidazole and clindamycin (2 weeks)
3	*S aureus*	Rifampicin and flucloxacillin (2 weeks), then doxycycline (6 weeks)
4	*S aureus*	Rifampicin and flucloxacillin (7 weeks)
5	Skin flora only	Vancomycin and rifampicin (2 weeks), then flucloxacillin and fusidic acid (2 weeks)
6	Coliform	Rifampicin and clindamycin (9 weeks)
7	*Proteus* spp	Flucloxacillin (3 weeks), then piperacillin/tazobactam (1 week), then vancomycin and rifampicin (2 weeks)
8	Coagulase-negative *Staphylococcus*	Flucloxacillin (10 weeks)
9	*S aureus*	Rifampicin and flucloxacillin (4 weeks)
10	MRSA	Vancomycin, rifampicin, gentamicin, mupirocin and chlorhexidine (2 weeks), then doxycycline and rifampicin (4 weeks)
11	Coliform	Meropenem (2 weeks), then ertapenem (5 weeks)
12	*Enterococcus* spp	Flucloxacillin, amoxicillin (10 weeks) and gentamicin (first 2 weeks only)
13	Coliform and *Klebsiella pneumoniae*	Rifampicin and flucloxacillin (6 weeks)
14	*S aureus* and Group A *Streptococcus*	Co-amoxiclav (2 weeks)
15	MRSA and *Enterococcus* spp	Flucloxacillin and gentamicin (2 weeks), then clindamycin (2 weeks), mupirocin and chlorhexidine (thoughout)
16	*S aureus* and *Enterococcus* spp	Rifampicin, metronidazole and clindamycin (2 weeks)

## Discussion

The management of implant-related infection is a controversial topic. The conventional method of treating these infections is serial surgical debridements, culture specific antibiotic treatment and removal of the implants. There is a distinction made between early (<8 weeks) and late onset infections (>8 weeks), with many advocating retention of the implants in early cases but removal in late cases.^4^ Treatment of infection in implants used for fracture stabilisation is further complicated by the fact that skeletal stability will be lost if implants are removed prior to fracture union.^5^

Berkes *et al* have shown a 71% fracture union rate following early postoperative wound infection using the standard techniques described above and implant retention.^5^

The infective organism is important, with different cure rates expected for different organisms. *S aureus* is more virulent than most and will often present acutely whereas coagulase-negative *Staphylococcus* often presents later with a more indolent clinical presentation.^6^ Similar to the literature, our series showed nine different infective organisms with the most common being *S aureus*.

The antibiotics used in the treatment of implant-related infection have been studied extensively over many years. The main target for these studies has been *S aureus* owing to it being the most common infective organism although the most important factor when deciding on the choice of antibiotics is the microbiological cultures. Rifampicin has been shown to be one of the most effective antibiotics, especially when used in combination with daptomycin.^7^

Biofilm formation is an important consideration with any infected implant. Biofilms are characterised by microcolonies of bacteria encased in a protective extracellular polymeric matrix. They can develop on any abiotic device implanted into a living organism. Given that one of the most common types of device implanted is a joint arthroplasty, most of the research has been carried out in orthopaedics. Currently, there is no widely accepted treatment for biofilms other than removal of the infected implant.^8,9^

Much of the known orthopaedic experience of NPWT dressings has been with their use in children who develop a deep spinal infection following spinal fusion and instrumentation.^10–12^ Most of these operations were carried out in children who had cerebral palsy, spina bifida or neuromuscular scoliosis. Removal of the metalwork in these children would be associated with significant complications, and many novel techniques have therefore been tried in this specialised subset of patients to try and cure the infection while still retaining the implants. Using VAC^®^ therapy, Canavese and Krajbich report that they were able to retain the metalwork in 32/33 patients, and cure the infection in 30/33 patients^12^ while van Rhee *et al* stated there were no signs of chronic infection in 6/6 neuromuscular scoliosis patients using VAC^®^ therapy and retaining the metalwork.^11^

A few other studies have looked at NPWT in infection associated with arthroplasties and a variety of other orthopaedic metal implants.^12–14^ One paper reported the use of VAC^®^ dressings in spinal cord injury patients who developed deep wound infections following spinal instrumentation.^14^ The authors had a 100% success rate in covering the metalwork but had only used it on two patients. Another paper looked more specifically at early hip joint infections following arthroplasty.^13^ There was a resolution of the infection in 26/28 patients, with a mean duration of treatment of 9 days (range: 3–16 days) and a mean follow-up of 36 months. Lehner *et al* reported on their experience of NPWT with instillation, and showed a cure rate of >80% with retention of the implants in both acute and chronic infections.^15^

Overall, these papers have shown an 80–100% success rate in curing the infection while retaining the implant. Our case series showed an overall success rate in curing the infection and retaining the implant in 16/16 patients, who survived to one year. This is similar to most of the other published series on the subject.

The novel aspects of this modified method are the use of the polyvinyl alcohol foam and irrigation under suction, which prolongs the life of the foam and facilitates effective debridement of infected tissue at regular intervals under GA. The method seems to improve blood supply to the wound and makes it possible to distinguish easily between vital (granulation) and non-vital tissue during debridement. The wound size decreases from treatment to treatment and skin closure helps to provide an effective barrier to further infection as well as also minimising wound contracture, which may otherwise necessitate further procedures such as skin grafts or skin flaps.

The main disadvantage of the treatment is the fact that inpatient treatment may require a lengthy stay with continuous irrigation and suction. The patient will require at least three visits to theatre under GA, which may not be appropriate if the patient has significant medical co-morbidities. There is also the added cost of the NPWT equipment and the nursing staff on the ward may require extra training on NPWT. However, as described above, the treatment of infected metalwork poses a significant clinical challenge and the modified technique provides an alternative adjunct for successfully retaining metalwork in these complex patients.

There are some limitations with our study, which included the small sample size, the short follow-up period of one year and the heterogeneous range of conditions included in the study.

## Conclusions

Patients with infected metalwork are a heterogeneous group, and often suffer high morbidity and mortality. NPWT has already shown potential in the treatment of orthopaedic patients with infected metalwork, particularly in extremity cases. This modified method provides an adjunct to standard NPWT and may provide a valuable option for the treatment of orthopaedic patients with infected metalwork.
